# Persistence of Multimorbidity Among Women Aged 15–49 Years in India: An Analysis of Prevalence, Patterns and Correlation

**DOI:** 10.3389/ijph.2021.601591

**Published:** 2021-05-19

**Authors:** Babul Hossain, Dipti Govil, Md Illias K. Sk

**Affiliations:** International Institute for Population Sciences, Mumbai, India

**Keywords:** younger women, Multimorbidity, Socio-economic pattern, coexisting morbidities, overweight and hypertension

## Abstract

**Objectives:** The present study has examined the patterns and possible correlates of coexisting morbidities among women aged 15–49 years based on biomarker measurement data at the national level in India.

**Methods:** National Family Health Survey conducted during 2015–16 used in the present study. Simple disease count approach was used to calculate the multimorbidity among women. Multinomial logistic regression was applied to analyze the predictors of multimorbidity among women.

**Results:** Almost 30% of the women had any of the selected morbidity and 9% of them had two or more morbidities. Hypertension and overweight combination (3%) was the most prevalent among women. The risk of having two or more morbidities was predominantly high among women aged above 30 years, low educated women, women from the wealthier group, ever-married women and women who were consuming tobacco as compared to their counterparts.

**Conclusions:** From the policy perspective, the identification of groups of women vulnerable to multimorbidity will help in the selection of programmatic focus and preventive public health intervention in adult phase to reduce the multimorbidity burden among women in old ages.

## Introduction

### Background

Multimorbidity has been defined as the coexistence of two or more chronic physical or mental or mixed health condition within an individual [1, 2]. Previous studies have found that the prevalence of multimorbidity varies between 10% and 60% in developed countries [3, 4]. A study from low-middle income countries also showed that one out of four persons have multimorbidity [5]. An individual with multimorbidity has higher odds of mortality [6]. Multimorbid persons are more dependent on health facilities, medication and face substantial economic burden where health care provider and medicine constituted the largest share of out of the pocket expenditure [7, 8]. Multimorbidity not only affects physical and mental functioning but also results in a negative impact on satisfaction with the self-reported health of an individual [9, 10]. multimorbidity influences the workload among women and increases care-seeking behavior [11, 12].

Previous studies have documented that there is a strong association between the multimorbidity and socio-economic status (SES). It has been found that the prevalence of multimorbidity among low educated adults is equivalent to highly educated adults who were at least ten to fifteen years older than them [13]. A similar scenario can be seen in developing countries [6, 13]. Studies from developing and developed countries also show a higher prevalence of multimorbidity among adults belonging to lower income group and underprivileged section in the countries [14–16]. Studies have documented that risky health behaviors like smoking, alcohol consumption are found to be associated with multimorbidity condition [14, 15]. With the increase in age, the prevalence of multimorbidity is expected to increase [3, 4, 6]. However, recent studies have concluded that the absolute number of individual with multimorbidity is higher among the population younger than 60 years. It has been demonstrated that during the adolescent phase, the prevalence of multimorbidity is relatively high [16]. In the other hand, sex acts as a moderator in the occurrence of multiple chronic conditions. A large body of evidence suggests that female are more prone to suffer from multiple coexisting health problems than male [7, 9, 15].

### Objectives

In the Indian context, studies in these issues are limited. Major concerns of the previous studies were aged population, or the patients attending primary care based on self-reported data or estimates were limited for the particular study area in India [7, 8]. Specific knowledge on multimorbidity for women aged 15–49 is missing despite we know that this group of women has high magnitude of multimorbidity in later ages. The present study assessed the pattern and possible correlates of co-presence of selected morbidities among women aged 15–49 years based on biomarker measurement data at the national level in India.

## Methods

### Data Source

Data from the National Family Health Survey-4 (NFHS-4) were used for the present study. The NFHS is a large scale multi-round survey, conducted throughout the country in 2015–16. The Ministry of Health and Family Welfare (MOHFW), Government of India, designated the International Institute for Population Sciences (IIPS) Mumbai, as the nodal agency, responsible for providing coordination and technical guidance for the survey. This nationally representative survey is an Indian version of the Demographic and Health Survey (DHS), providing consistent and reliable estimates of fertility, mortality, family planning, child nutritional status, morbidity, utilization of maternal and child health care services, anemia, utilization and quality of health and family planning and other related indicators at the national, state and regional levels. The NFHS-4 covered a nationally representative sample of 601,509 households, comprising 699,686 women of reproductive age (aged 15–49 years). The survey also provided data on 103,525 men aged 15–54 years and 259,627 under five children [17].

### Participants

In our study, the dependent variable was morbidity condition among the women aged 15–49 years. The selected sample for this study included women with valid biomarker measurement data. After excluding the missing values, the study used a total sample of 656,080 women [17].

### Variables

#### Outcome Variables

The variable of interest was morbidity categorized with no morbidity (zero health condition present), one morbidity (with one health condition) and multimorbidity (two or more health conditions). The study included four health conditions viz. anemia, hypertension, high glucose level, and overweight [18]. Demographic and Health Surveys (DHS) cut-off were applied to define morbidities [17]. Hypertension was measured using the blood pressure level (measured in mmHg). Women were considered having hypertension if they had a systolic measure of more than 139 mmHg, and the diastolic measure was more than 89 mmHg. Women having a random glucose level of more than 160 mg/dl were considered having high glucose condition. Overweight was measured using BMI level (as weight, measured in kilograms divided by height squared meters). The woman was considered overweight if women’s BMI was more than 25.0 kg/m^2^. Anemia was measured using the hemoglobin level (grams/dl). Women with hemoglobin level in the blood less than 7 g/dl were considered as anemic. Simple disease count approach was used to measure multimorbidity. The categorical variable, health condition was created from the combinations of anemia, hypertension, high glucose level, overweight.

#### Independent Variables

After an extensive literature review, several independent variables were considered for the study. The socio-economic characteristics at the household level: the place of residence, religion, wealth quintile, caste, and region were considered. Place of residence was grouped into two categories; rural and urban. Religion status was divided into four categories Hindu, Muslim, Christian and others. Wealth quantiles (based on scores on kinds and number of goods owned and consumed by the households like T.V. any locomotor, housing characteristics like flooring materials drinking water type, toilet facility *etc*.) divided into quintiles: poorest, poorer, middle, richer, richest. Caste status was categorized into *Scheduled caste* or S.C./*Scheduled tribe* or S.T. *Other backward* class or OBC, Others. The present study included the following indicators at the individual level; Age, marital status, educational level, alcohol and tobacco consumption. The age group of women were divided into four groups: 15–19, 20–29, 30–39 and 40–49. Marital status was categorized into: never married, currently married, widowed/divorced/separated. Educational level was considered for four groups: no education, primary, secondary and higher. The use of tobacco and alcohol by the women were considered as a health risk covariate in the study.

### Statistical Analysis

The analysis was restricted to 656,080 respondents due to 6% missing data on the health condition. For the univariate analysis, the percentage distribution of the sample was calculated. For the bivariate analysis, the number of health condition by the different socio-economic background was calculated. The prevalence of multimorbidity was calculated for different age groups. As our outcome variable was in three categories, multinomial logistic regressions were applied, including all the exposure variables in the same model. Results were reported as adjusted relative risk ratios (RRR) with 95% CI. The analyses were done using STATA 15.1.

## Results

We analyzed data from 656,080 respondents aged 15–49 years (Table 1). One-third of the women were in 20–29 years of age group. 47.2% of women had secondary education, and very few had higher education (12.6%). The majority of women were currently married (73.6%) and lived in rural areas (66.2%). 6.2% of the women had used tobacco, whereas 1.2% of the respondents had used alcohol.

**TABLE 1:** Health condition and background characteristics of women aged 15–49 in the study sample, India, 2015–2016.

	Sample characteristics	Health condition		
Variables	Total (N = 656, 080)	No morbidity (n = 400, 823)	Single morbidity (n = 198, 090)	Two or more morbidity (n = 57, 167)
Age group				
15–19	17.3	79.7	18.8	1.3
20–29	34	68.4	27.3	4.1
30–39	26.8	53.8	35.3	10.8
40–49	21.7	43.6	37.3	19
Level of education				
No education	27.7	59.4	31.3	9.1
Primary	12.5	58.5	31.2	10.2
Secondary	47.2	62.6	29	8.2
Higher	12.6	61.4	30.8	7.7
Place of residence				
Urban	33.8	53.8	34	12.1
Rural	66.2	64.7	28.2	6.9
Marital status				
Never married	22.4	77.6	20.3	2.1
Currently married	73.6	56.6	32.8	10.4
Widowed/divorced/separated	4.15	50.2	30.1	13.9
Wealth index				
Poorest	18	70.4	25.5	4
Poorer	19.8	67.3	26.9	5.4
Middle	20.7	62.1	29.7	8
Richer	21	55.5	32.7	11.6
Richest	20.5	51.4	35.2	13.3
Caste				
SC/ST	30.8	63.5	29.2	7.1
OBC	45.3	61.7	29.6	8.6
Other	23.8	57.2	32.2	10.5
Religion				
Hindu	70.7	61.6	29.9	8.3
Muslim	13.5	59.7	30.5	9.7
Christian	7.09	55.3	32.4	12.1
Other	4.71	57	32.4	10.4
Alcohol consumption				
No	98.8	61.1	30.1	8.7
Yes	1.2	57.8	33.3	9.2
Tobacco consumption				
No	93.7	61.3	30	8.6
Yes	6.24	58.7	32.8	9.1
Total		61.1	32.2	8.7

Source- Computed from NFHS, 2015–2016.

Notes: Analyses adjust for sampling weights, SC denotes Scheduled castes and ST denotes Schedule tribes, OBC denotes other backward classes.

Last three columns of Table 1 shows the percentage distribution of health condition by the different background characteristics. The prevalence of the multimorbidity among women was 8.7%, and 32.2% of the women had at least one chronic health condition. The prevalence of two or above morbidities increased with age (See Table 1). The prevalence of two or above morbidities was higher among women living in urban area (12.1%), belonging to the Primary educational group (10.2%) and higher quintile of wealth index (13.3%). Widowed/divorced/separated (13.9%) women and, women consuming alcohol (9.2%) and tobacco (9.1%) in any form had a higher share of multimorbidity.

**FIGURE 1:**
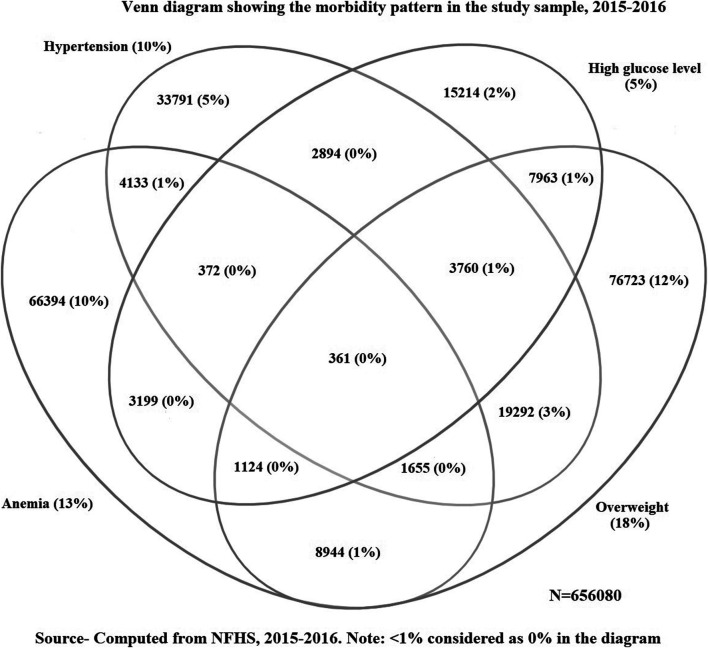
Venn diagram showing the morbidity pattern in the study sample, India, 2015–2016.

Figure 1 shows the morbidity pattern in women. High glucose level and overweight contributed highest in multimorbidity status. As shown in Table 2, the combination of hypertension and overweight had the highest prevalence (3%) followed by the combination of overweight and high glucose level (1.4%) for all age groups. The combination of anemia and overweight were found as a chronic health condition in 15–19 and 20–29 years age groups, whereas overweight and hypertension combination was the most prevalent health condition in women aged 30–39 and 40–49 years.

**TABLE 2:** Age specific morbidity pattern in the study sample, India, 2015–2016.

Combination	15–19 years (n = 113,683)	20–29 years (n = 223,404)	30–39 years (n = 176,268)	40–49 years (n = 142,725)	15–49 years (N = 656,080)
	No. of subject (%)	No. of subject (%)	No. of subject (%)	No. of subject (%)	No. of subject (%)
Anemia	14,609 (12.85)	30,871 (13.81)	22,510 (12.77)	18,144 (12.71)	86,134 (13.12)
Overweight	4,441 (3.90)	28,127 (12.59)	44,349 (25.15)	42,898 (30.05)	119,815 18.26)
High glucose level	2,410 (2.11)	6,618 (2.96)	10,763 (6.10)	15,094 (10.57)	34,885 (5.31)
Hypertension	3,149 (2.76)	11,596 (5.19)	21,625 (12.26)	29,884 (20.98)	66,254 (10.09)
Anemia + overweight	426 (0.38)	2,970 (1.33)	3,752 (2.13)	3,260 (2.28)	10,409 (1.59)
Anemia + high glucose level	381 (0.34)	838 (0.38)	921 (0.52)	1,064 (0.75)	3,205 (0.49)
Anemia + hypertension	271 (0.24)	895 (0.4)	1,140 (0.65)	1,666 (1.17)	3,974 (0.63)
Overweight + high glucose level	152 (0.13)	1,448 (0.65)	3,545 (2.01)	5,398 (3.78)	10,544 (1.61)
Overweight + hypertension	225 (0.20)	2,476 (1.11)	6,944 (3.94)	9,862 (6.91)	19,506 (2.97)
Hypertension + High glucose level	54 (0.05)	300 (0.13)	780 (0.44)	1,612 (1.13)	2,476 (0.44)
Anemia + overweight + Hypertension	-	188 (0.08)	657 (0.37)	1,035 (0.73)	1898 (0.25)
Anemia + High glucose level + hypertension	-	-	148 (0.08)	188 (0.13)	369 (0.06)
Overweight + High glucose level + hypertension	-	185 (0.08)	1,165 (0.66)	2,712 (1.90)	4,077 (0.62)
Anemia + High glucose level + Hypertension + overweight	-	-	-	337 (0.24)	435 (0.06)

Source- Computed from NFHS, 2015–2016.

Note: No. of subject include only those women suffering the specific combination of health conditions. Combinations are calculated based on the simple counting approach. Analyses adjusted for sampling weights. “-“ denotes less than 0.05% prevalence.

Table 3 describes the association between multimorbidity and socio-economic status. The analysis shows that age, place of residence, level of education, caste, wealth index (economic condition), marital status, tobacco consumption were significantly explaining the single and multimorbidity among women. The RRR of single morbidity, when compared with no morbidity, was 2.61 times (95% CI 2.51–2.72) while RRR of multimorbidity when compared to no morbidity was 14.94 times (95% CI 13.5–16.5) among women aged 40–49 years. The RRR of single and multimorbidity compared to no morbidity were 1.16 times (95% CI 1.14–1.19) and 1.31 times (95% CI 1.25–1.34) for those women living in the urban area. The RRR of single and multimorbidity decreased with higher educational level. The single and multimorbidity were both found to be highest among women belonged to the wealthiest wealth index. The RRR of having single and multimorbidity increased among women who were widowed/divorced/separated. Women belonging to religion other than Hindu had a higher relative risk of single and multimorbidity. By the caste of the women, OBC category women were 0.92 times (95% CI 0.88–0.95) less likely to have multimorbidity as compared to SC/ST women. The result showed that those women using tobacco had 1.04 (95% CI 1.01–1.07) time of single and 1.91 (95% CI 1.86–1.97) times of risk of multimorbidity.

**TABLE 3:** Relative risk ratio (RRR) showing the effect of background characteristics on the single and multimorbidity among women, India, 2015–2016.

Background characteristics	Single morbidity			Multi morbidity		
	Adjusted RRR	CI		Adjusted RRR	CI	
		LL	UL		LL	UL
Age group						
15–19^®^						
20–29	1.29***	1.24	1.33	2.29***	2.08	2.53
30–39	2.00***	1.92	2.07	6.82***	6.16	7.54
40–49	2.61***	2.51	2.72	14.94***	13.50	16.54
Level of education						
No education^®^						
Primary	1.08***	1.05	1.11	1.21***	1.15	1.27
Secondary	1.07***	1.04	1.09	1.14***	1.09	1.19
Higher	0.98	0.94	1.01	0.86***	0.80	0.91
Place of residence						
Rural^®^						
Urban	1.16***	1.14	1.19	1.31***	1.25	1.34
Marital status						
Never married^®^						
Currently married	1.45***	1.40	1.50	1.93***	1.79	2.09
Widowed/divorced/separated	1.48***	1.40	1.56	1.95***	1.77	2.15
Wealth index						
Poorest^®^						
Poorer	1.07**	1.04	1.10	1.41***	1.34	1.49
Middle	1.23***^a^	1.19	1.26	2.01***	1.90	2.11
Richer	1.45***	1.41	1.50	3.03***	2.86	3.21
Richest	1.66***	1.61	1.73	3.67***	3.44	3.92
Caste						
SC/ST^®^						
OBC	0.92***	0.90	0.94	0.92***	0.88	0.95
Other	1.00*	0.99	1.05	1.07**	1.02	1.12
Religion						
Hindu^®^						
Muslim	1.09***	1.06	1.12	1.29***	1.24	1.36
Christian	1.02	0.96	1.08	1.17**	1.06	1.28
Other	1.11**	1.05	1.15	1.22***	1.13	1.31
Tobacco consumption						
No^®^						
Yes	1.04**	1.01	1.07	1.91**^b^	1.86	1.97
Alcohol consumption						
No ^®^						
Yes	1.07	1.01	1.14	1.06	1.00	1.13

Source- Computed from NFHS, 2016–2016, UL and LL denotes upper limit and lower limit.

Note: *P < 0.10, ** < 0.05, and *** < 0.01 level of significance, ® denote Reference category, Base outcome = No morbidity.

## Discussion

The study analyzed multimorbidity condition among women aged 15–49 years of age using biomarker measurement data in India. The overall prevalence of multimorbidity was 8.7% in the study population. The study explored that overweight and hypertension contributed more to multimorbidity conditions. Age, place of residence, level of education, caste, wealth index (economic condition), marital status, tobacco consumption were significantly associated with multimorbidity condition, whereas alcohol consumption was not found to be associated with multimorbidity condition in women.

The estimation of multimorbidity in the present study was lower compared to other studies. A study based on global aging and adult health (SAGE) data for India along with other five low and middle-income countries (LMIC) showed that in 18–49 age group for both male and female, the prevalence of multimorbidity was 12% [5]. This study was on the general population and included eight self-reported health condition. Whereas, a state-based study in India targeting patients attending primary care observed the higher prevalence of the multimorbidity among women and for 18–49 age group [7, 8]. Although the study by Prados-Torres (2012) in Spain found that women had a little higher (13%) prevalence of multimorbidity for 15–44 years age group which is anomalous to our findings [19]. The lower level of estimation of multimorbidity in the present study may be because of the limited number of health condition.

Along with existing studies, the present study also found that multimorbidity condition is inversely associated with education level. The German-based study reported that low educated middle-aged women were more likely to develop multimorbidity [4].The probable explanation may be due to knowledge and awareness related to access to health services [21]. Women with lower education may have a lack of knowledge about health management. The present study found a significant risk of multimorbidity among the women residing in urban areas and belonged to higher wealth index. Although we did not find any association between multimorbidity and alcohol consumption in women in India, it was evident that lifestyle-based factors like smoking and alcohol abuse among urban women caused a higher risk of NCDs compared to women residing in rural areas [17, 25]. The previous study found a lack of physical activity among women who belonged to the middle to higher household income and had a higher prevalence of metabolic syndrome that further lead to multimorbidity [20]. The present study also found that ever married were having a higher prevalence of the multimorbidity compared to never-married women [24]. A cross-country study using SAGE database found Ghana, India, South Africa, and Spain also support the present study findings [5, 21]. Previous studies support the present study finding that women who were tobacco user had a significantly higher risk of multimorbidity [22]. This study found no significant association between alcohol consumption and multimorbidity. However, many studies had found a strong association between alcohol consumption and multimorbidity [23]. The probable explanation may be lower alcohol consumption among women compared to men in Indian settings [17].

## Strengths and Limitations

The strength of the present study is that the estimations are based on nationally representative biomarker measurements for women aged 15–49 years of age, following the standard cut off to define the morbidity condition. This aspect allows the generalization of the findings with other countries. The study exclusively focused on younger women on the issue of Multimorbidity in India, which is rarely being reported in previous studies.

However, our study has several limitations. The study only included four health conditions to measure multimorbidity among women. The study included a limited number of morbidities, and that may lead to the underestimation of multimorbidity. In the present study, the selected health conditions were different risk factors rather than the actual disease. The risk factors give a concerning awareness of possible illness in the future rather than the actual burden of diseases [18]. The definition of health conditions does not include drug treatment assumptions. Thus, it may underestimate the prevalence of morbidities, especially for hypertension. The present study aims to highlight the associative multimorbidity (only statistically associated, not known to be causal). The causal relationship between coexisting morbidity was not explored here. The severity of morbidity combination is not measured. Furthermore, studies are needed to understand the causal multimorbidity among adult women in India. There is a need for studies considering greater number of morbidity conditions among young women to better understand the multimorbidity situation.

## Conclusion

This present study contributes to understand the multimorbidity among adult women aged 15–49 years. The study identified overweight and hypertension as the most critical combination among adult women. The more vulnerable women were less educated, widow/divorced/separated, and tobacco consumers. Hence, it is essential to consider these factors while looking at health condition among women. From the policy perspective, the identification of groups of women vulnerable to multimorbidity will help in the selection of programmatic focus and preventive public health intervention in adult phase to reduce the multimorbidity burden among women in old ages. It drives toward achieving sustainable development goal-3 about healthy lives and wellbeing for all.

## Data Availability

The original contributions presented in the study are included in the article/Supplementary Material, further inquiries can be directed to the corresponding author.
